# Family physicians’ views on participating in prevention of major depression. The predictD-EVAL qualitative study

**DOI:** 10.1371/journal.pone.0217621

**Published:** 2019-05-30

**Authors:** Patricia Moreno-Peral, Sonia Conejo-Cerón, Anna Fernández, Carlos Martín-Pérez, Carmen Fernández-Alonso, Antonina Rodríguez-Bayón, María Isabel Ballesta-Rodríguez, José María Aiarzagüena, Carmen Montón-Franco, Michael King, Irwin Nazareth, Juan Ángel Bellón

**Affiliations:** 1 Instituto de Investigación Biomédica de Málaga (IBIMA), Málaga, Spain; 2 Distrito Sanitario Málaga-Guadalhorce, Málaga, Spain; 3 Red de Investigación en Actividades Preventivas y Promoción de la Salud (redIAPP), Barcelona, Spain; 4 Parc Sanitari Sant Joan de Déu, Fundació Sant Joan de Déu, Barcelona, Spain; 5 CIBERESP, Centro de Investigación Biomédica en Red de Epidemiología y Salud Pública, Barcelona, Spain; 6 Centro de Salud Marquesado, Servicio Andaluz de Salud, Granada, Spain; 7 Service Assistance Programs, Regional Health Management Valladolid, Valladolid, Spain; 8 Centro de Salud San José, Servicio Andaluz de Salud, Linares, Jaén, Spain; 9 Centro de Salud Federico del Castillo, Servicio Andaluz de Salud, Jaén, Spain; 10 Centro de Salud San Ignacio, Primary Care Research Unit, Osakidetza, Bilbao, Spain; 11 Centro de Salud Casablanca, Zaragoza, Spain; 12 Aragonese Institute of Health Sciences, IIS Aragón, Zaragoza, Spain; 13 Departamento de Medicina y Psiquiatría, Universidad de Zaragoza, Zaragoza, Spain; 14 Division of Psychiatry (Faculty of Brain Sciences), University College London (UCL), London, United Kingdom; 15 PRIMENT Clinical Trials Unit, University College London (UCL), London, United Kingdom; 16 Department of Primary care and Population Health, University College London (UCL), London, United Kingdom; 17 Centro de Salud El Palo, Servicio Andaluz de Salud, Málaga, Spain; 18 Departamento de Salud Pública y Psiquiatría, Universidad de Málaga, Málaga, Spain; Newcastle University, UNITED KINGDOM

## Abstract

**Background:**

The predictD intervention, a multicomponent intervention delivered by family physicians (FPs), reduced the incidence of major depression by 21% versus the control group and was cost-effective. A qualitative methodology was proposed to identify the mechanisms of action of these complex interventions.

**Purpose:**

To seek the opinions of these FPs on the potential successful components of the predictD intervention for the primary prevention of depression in primary care and to identify areas for improvement.

**Method:**

Qualitative study with FPs who delivered the predictD intervention at 35 urban primary care centres in seven Spanish cities. Face-to-face semi-structured interviews adopting a phenomenological approach. The data was triangulated by three investigators using thematic analysis and respondent validation was carried out.

**Results:**

Sixty-seven FPs were interviewed and they indicated strategies used to perform the predictD intervention, including specific communication skills such as empathy and the activation of patient resources. They perceived barriers such as lack of time and facilitators such as prior acquaintance with patients. FPs recognized the positive consequences of the intervention for FPs, patients and the doctor-patient relationship. They also identified strategies for future versions and implementations of the predictD intervention.

**Conclusions:**

The FPs who carried out the predictD intervention identified factors potentially associated with successful prevention using this program and others that could be improved. Their opinions about the predictD intervention will enable development of a more effective and acceptable version and its implementation in different primary health care settings.

## Introduction

Interventions to prevent depression are effective but their effect sizes are small to moderate [1–2]. These interventions are primarily psychological or educational and are provided by mental health professionals [2]. Several randomized controlled trials of primary prevention of depression in primary care have been undertaken [3], although in only two the intervention was implemented by family physicians (FPs). In a third trial, (the "CATCH-IT" study) [4] the effectiveness in adolescents of brief counseling was compared with a motivational interview administered by FPs. While both interventions seemed effective, there was no evidence either way for superiority. Our research team carried out the predictD-CCRT study to compare a bio-psycho-social intervention implemented by FPs (the predictD intervention) versus usual care [5]. The predictD intervention is based on a risk algorithm to predict the onset of major depression at 12 months in primary care attendees [6]. This intervention was tailored to each patient based on his/her risk profile for depression (risk factors present) and his/her risk level (likelihood of becoming depressed at 12 months), and it was developed as five a priori active components [5–7]: a training workshop for FPs; communicating the level and profile of risk of depression to patients every six months in a 10- to 15-minute interview; constructing a personalized bio-psycho-social intervention to prevent depression; offering a brochure; and activating and empowering patients. This intervention reduced the incidence of major depression at 18 months follow-up by 21% versus the control group (usual care) [7] and it was also cost-effective [8]. Qualitative methodology is the best way to elucidate the active ingredients/mechanisms of action of these complex interventions in order to promote them and to adapt the interventions for application in different settings [9]. Therefore, the aim of our study was to assesss FPs’ opinions about the potential successful components of the predictD intervention for the primary prevention of depression in primary care and to identify areas for improvement.

## Methods

All participants gave written informed consent and participant anonymity was maintained using personal codes in the transcripts. This study was approved by the ethic committee: Ethics and Research Committee of Primary Health District of Málaga.

### Design

A qualitative study using face to face, semi-structured interviews was undertaken. Our ontological position is relativism (i.e. the view that reality is subjective and may differ from person to person). Our main aim is to understand how FPs have experienced the implementation of the predictD intervention, from their individual´s perspective, taking into account the context in which it had been implemented. The methodological approach used was phenomenology.

### Setting

The study population comprised FPs working at 35 urban primary care centres in seven Spanish cities (Barcelona, Bilbao, Zaragoza, Valladolid, Jaén, Granada and Málaga). The Spanish National Health Service provides universal coverage for citizens through a public system financed by taxes and is free at the point of use. Health care services are distributed into Health Areas and Basic Health Zones according to geographical, epidemiological and socioeconomic criteria. Each Health Area covers a population of 200,000–400,000 inhabitants and is composed of several Basic Health Zones, which are the minimum units of health care organization. Basic Health Zones are organized around a primary care centre. Primary care centres serve populations of between 5,000 and 30,000 inhabitants and the primary care teams are composed of FPs, pediatricians, nurses and, in some cases, social workers. They provide a broad range of services, including the treatment of common mental disorders (shared with mental health specialists in severe cases) such as anxiety or depression [10], health promotion and preventive services. In Spain, primary care is the patient’s first point of contact with the public health system. Each patient is assigned to only one FP, who functions as a gatekeeper to the wider system. Patients can visit their FP as often as they want without having to pay for consultations, even when they do so for preventive reasons. The average time that a Spanish FP spends per patient is between 8 and 10 minutes.

All the primary care centre staff members, including the GPs, are salaried. GP salaries contain two elements: a larger fixed payment and a smaller incentive, based on elements such as numbers of patients assigned, fulfilment of objectives, patterns of prescription and pay-for performance incentives [11].

### Sample selection

For inclusion, the FP had to be one of the 70 FPs who had performed the predictD intervention and completed the follow-up at 18 months.

### Procedure

The interviewers were experts in qualitative interview techniques. Research assistants contacted the FPs who were informed of the date, time and place of the meeting but did not explain the study objective in detail. The FPs were only told that the purpose of the meeting was to seek their views on their participation in the predictD-CCRT study. None of the FPs knew the results of the effectiveness [7] or cost-effectiveness [8] of the predictD intervention when they were interviewed. The interview guide was piloted with one FP. The topic guide used in the interviews is shown in Table 1. The interviews were conducted between March and April 2013 and lasted 20–60 minutes. The interviews were all conducted in a place convenient to the FPs (quiet areas of primary care centres) and were audio-recorded digitally, transcribed and anonymized.

**Table 1:** The interview guide.

Topic	Guide questions
Experience with participation in the project	How was your experience participating in the predictD project?
Provide information regarding the likelihood of becoming depressed in the future	Based on your experience, what is your opinion on giving your patients information about their likelihood of becoming depressed in the future? Did you have difficulty informing your patients about their risk of becoming depressed? What is your opinion regarding the frequency with which you informed your patients of the risk of depression (every 6 months)? It is possible that in some of your patients you may have tried to comment on their risk factors for depression. If so, what was your experience like? Do you think some of your patients may have been emotionally affected by being informed about their risk of becoming depressed? If so, could you tell me about this? Do you think that giving your patients information about their risk of becoming depressed affected you emotionally as well? If so, could you tell me about this? Would you change something about the content or the way you informed your patients about their risk of becoming depressed?
Advice and Brochures	It is possible that during the interviews with your predictD intervention patients you may have tried to advise them to prevent depression. What is your opinion of this? What do you think of the brochure’s usefulness in preventing depression? What tips do you find most helpful in the brochure? Would you change anything in the brochure?
Usefulness of the predictD intervention	What do you think of the predictD intervention in terms of its usefulness in preventing depression in primary care patients? What ingredients or parts of the predictD intervention do you think are most helpful in preventing depression? What ingredients or parts of the predictD intervention do you think are least helpful in preventing depression? Regarding the predictD intervention you have implemented, what has been most useful to you as a professional or for your practice activity? Regarding the predictD intervention you have implemented, what has been most useful for your patients?? Do you think the intervention is more effective for a given patient profile? Which profile? Do you believe that the predict intervention should not be implemented for a given patient profile?
Interaction	Did the relationship with any of your patients change at all after you informed them of their possibility of becoming depressed in the future? If so, in what way or how did it change?
Perception of effectiveness	Do you think the predictD intervention you have implemented for your patients has been effective in preventing depression? To what extent?
Difficulties	What barriers or difficulties have you encountered in developing and implementing the predictD intervention?
Changes and improvements	If you were to repeat the predictD intervention with your patients, what would you change?
Future applicability	In the event that the predictD intervention is effective, what do you think about its widespread future applicability as a prevention program? Is there anything about the predictD intervention you are currently employing with your patients? What and why? If the predictD intervention were to be widely applied in the future, would you recommend that your family and friends participate in this prevention program? Can you think of a useful strategy for the future implementation of this intervention as a prevention program?
Intervention training	You will remember that before you started the predictD intervention you participated in a predictD intervention training workshop. What is your opinion of the predictD intervention training workshop? What do you think of the contents of the training workshop for the predictD intervention? What do you think of the teaching method used to deliver the course for the predictD intervention? What is your opinion about the duration of the training workshop for the predictD intervention? What would you change about the training workshop for the predictD intervention? We are now finishing the interview. Would you like to add anything else?

### Analysis

We used thematic analysis [12]. Using the transcriptions and to ensure data quality, the information obtained was triangulated by the participation of three analysts (PMP, AF, SCC) from different cities and professional backgrounds with experience in using qualitative research who independently analyzed the interviews [13]. These three researchers became familiar with the interviews by listening, reading and rereading them. Themes were identified and manually coded independently by each of the three researchers involved in the analyses. They then came together to compare and discuss differences in the analyses. Themes were then recoded and classified, identifying common patterns and convergences and divergences in the data through a process of constant comparison.

In order to enhancing the quality of this research [13–14], the interviewers and analysts kept a personal research diary alongside the data collection and analysis to record any reactions to events occurring during the research. Respondent validation was conducted, comparing analysts’ interpretation of the phenomena with that of those who had participated. Participants were sent a summary of the findings. Forty FPs participated in this validation. Changes suggested by the FPs were incorporated into the final description of the phenomena.

### Ethics statement

All participants gave written informed consent and participant anonymity was maintained using personal codes in the transcripts. The study was approved by the ethics committees in each participating Spanish city.

## Results

The 70 FPs participating in the intervention arm of the predictD-CCRT study were invited to take part in the predictD-EVAL study. Of these, 67 (95.7%) agreed to participate. The remaining three did not participate because they did not complete the predictD-CCRT study: two due to sick leave and the third due to relocation to another health centre. The characteristics of the FPs are shown in Table 2.

**Table 2:** Sociodemographic and professional characteristics of the family physicians interviewed (N = 67).

Characteristic	Number	Percentage (%)
**Gender**		
Female	37	55.2
**Mean age (range)**	52.44 (34 to 62)	
**City**		
Málaga	10	14.9
Jaén	10	14.9
Granada	9	13.4
Zaragoza	9	13.4
Valladolid	10	14.9
Bilbao	10	14.9
Barcelona	9	13.4
**Size of town where practice is located**^a^		
2.500–30.000 inhabitants	13	19.4
30.001–200.000 inhabitants	11	16.4
>200.000 inhabitants	41	61.2
**Family physicians list size average (range)**^b^	1540.5 (805 to 2150)	
**Average time spent per patient** ^c^		
3–6 minutes	10	14.9
7–9 minutes	31	46.3
≥10 minutes	25	37.3
**Relationship with mental health team**^d^		
Very poor/poor	8	11.9
Average	23	34.3
good/very good	34	50.7
**Satisfaction with support from mental health team**^e^		
Very dissatisfied/dissatisfied	14	20.9
Neither dissatisfied nor satisfied	23	34.3
Satisfied/very satisfied	29	43.3
**Comfort handling antidepressants**^f^		
Uncomfortable	3	4.5
Neither uncomfortable nor comfortable	15	22.4
Comfortable/Very comfortable	48	71.6
**Relationship with primary care nurses**^g^		
Very poor/poor	2	3
Average	6	8.9
good/very good	58	86.6
**Relationship with primary care social workers**^h^		
No social worker	7	10.4
Poor	4	6
Average	13	19.4
Good/very good	42	62.7
**Low Extraversion (EPQR-A) (score of 0–4)**^i^	37	55.2
**Low Neuroticism (EPQR-A) (score of 0–4)**^j^	61	91
**Low Psychoticism (EPQR-A) (score of 0–4)**^k^	60	89.6
†**Style of professional practice**		
Mean job satisfaction (range)^l^	16.03 (9 to 20)	
Mean perception workload (range)^m^	14.1 (6 to 20)	
Biomedical versus psychosocial orientation (range)^n^	10.3 (4 to 19)	
**Long-term contract (yes)**^o^	62	92.5
**Accredited to train residents (yes)**^p^	37	55.2
**Training fourth-year resident (yes)**^q^	24	35.8
**Training first-year resident (yes)**^r^	16	23.9
Member of the communication & health group* **(yes)** ^s^	5	7.5

EPQR-A: Eysenck Personality Questionnaire Revised-Abbreviated (*Sandin B*, *Valiente RM*, *Chorot P*, *Olmedo M*, *Santed MA*. *Spanish version of the Eysenck Personality Questionnaire-Revised (EPQR-A) (I)*: *exploratory factor analysis [in Spanish]*. *Revista de Psicopatología y Psicología Clínica*. *2002; 7*:*195–205*.)

* Family physicians with extensive training in doctor-patient communication skills.

^a^ 2 missing;

^b^ 6 missing;

^c^ 1 missing;

^d^ 2 missing;

^e^ 1 missing;

^f^ 1 missing;

^g^ 1 missing;

^h^ 1 missing;

^i^ 4 missing;

^j^ 3 missing;

^k^ 3 missing;

^l^ 2 missing;

^m^ 2 missing;

^n^ 2 missing;

^o^ 2 missing;

^p^ 2 missing;

^q^ 3 missing;

^r^ 2 missing;

^s^ 2 missing

**†** Mira JJ, Llinás G, Gil V, Orozco D, Palazón I, Vitaller J. [Validation of an instrument for identifying styles of the professional practice of the primary care doctor]. Aten Primaria. 1998; 21:14–22.

The FPs’ opinions were described in six broad thematic categories.

1. Opinions on the tentative/exploratory components of the predictD intervention
  - Level of risk. Some doctors found the categories of low, moderate and high risk more useful than the percentage: *[FP60*: *It was more the high*, *moderate*, *adjective I could give them rather than a figure of 20 or 35]*.
  - Risk profile. In general, commenting on risk factors, especially if they are modifiable, is considered important and useful: *[FP47*: *I think that the most useful part is the detection of risk factors and that you can discuss to a certain extent where the fundamental risk factor is]*. Indeed, FPs perceived that this information was a good start to encourage a deep conversation about the emotional problems of their patients.
  - Frequency with which information on the level and profile of risk is received. In general, physicians consider six months to be an adequate period of time: *[FP45*: *This is enough time to see the progression*, *not less time though because there could be stressful life events]*. However, some consider it too long: *[*FP37: *There may be people whom I had almost forgotten and they were a little lost*, *so maybe a little less time]*. There were also opinions about adapting that frequency to the patient: *[FP43*: *…or providing special follow-up*…*to be a little more attentive to those patients who are at risk and in those patients perhaps in three months or perhaps eight months]*.
  - Brochure. The doctors had a positive opinion of the pamphlet: *[FP43*: *They (the patients) said*: *look at this*, *it is very good for me*…*for some of them it even helped them to go deeper*, *to look for more things*, *I think it was positive]*. However, they also have concerns about whether patients read and understand it: *[FP64*: *I honestly don’t know if they looked at it that much*…*]*.
  - Advice. The proposed recommendations were considered important and necessary. *[FP35*: *So when you make them think and give them some advice*, *I think that’s what they’ve been most grateful for]*. They perceived that it was useful to have it as a guide.
  - Training. In general, they found the training they received useful. *[FP45*: *The workshop we did was very interesting*…*it was very productive]*. They also pointed out the need for more extensive and continuous training: *[FP54*: *Maybe a little short*, *have a little more training*, *extend it a little more]*.
2. Strategies used by the FPs to successfully implement the intervention.
  - Spend more time with patients. [FP28: *I would schedule them for lengthier appointments because it allowed me time to develop a more intimate*, *deeper relationship with the patient]*. In this sense, it was important that the physician was able to manage their own agenda, in order to be able to schedule lengthier appointments. Some referred specifically to patients who had a greater likelihood of becoming depressed at 12 months: *[FP40*: *There was more risk*, *more emphasis needed to be placed on measures and such*, *in other words*, *a little more emphasis on the intervention]*.
  - Focusing on patients and using doctor-patient communication skills. The physicians undertook interventions adapted to the individual: *[FP43*: *It depends on what the patient wants to tell you*, *what he/she wants to do*…*you follow the script differently depending on each patient]*, empathizing with the patient: *[FP6*: *You empathize a little more*…*you try to understand their situation a little better and see it in another way]*, and using positive messages: *[FP40*: *It depends on how you say things*, *you always try to give a message more from a positive point of view]*.
  - Strengthen and implement problem-solving strategies. *[FP27*: *I take a more problem-solving approach to both low-risk and high-risk individuals]*.
  - Activate the resources available to each person. *[FP21*: *What I tried to do was to get them to analyze the problem and look for solutions…I tried not to give advice but to have them work on that process]*.
  - Involve social work colleagues. *[FP38*: *Some things did change because they were addressed*, *the social worker intervened]*.
  - Don’t get too involved. The FPs pointed out that they tried not to take the ownership of their patients´problems with the aim of not becoming emotionally over involved *[FP43*: *I try to understand the patient in his/her situation but I don’t make it my own]*.
3. Barriers perceived by FPs.
  - Communication and understanding. Some FPs had difficulty communicating the risk of depression to their patients: *[FP40*: *it was a little more difficult for me*, *I wasn’t really sure*…*I tried to find the right words to give a positive message regarding what it is]*. They also pointed out that the patients themselves had trouble understanding the risk of becoming depressed: *[FP54*: *Sometimes they didn’t quite understand what it was to be at risk for depression]*.
  - Time and workload. *[FP40*: *time is fundamental…there is not enough time to be even minimally educated]*. *[FP28*: *It generated a little more work for me but I think it was worth it]*. They commented that during the study patients had more frequent visits: *[FP40*: *I had the feeling that they came more often*…*]*. However, other physicians pointed out that it did not involve much effort or a substantial increase in work activity: *[FP14*: *The individuals we had in this protocol did not attend more*, *nor did they have increased demand]*. *[FP22*: *It wasn’t an extra effort for me*. *I’ve integrated it into the practice]*. Some FPs even pointed out that in the long term it could reduce the frequency of visits: *[FP36*: *They have neither become more demanding*, *nor have they attended more often*, *perhaps the other way round*…*By highlighting and bringing to light what was there*, *in one specific woman*…*it has led to longer intervals between consultations*, *because she has also improved]*.
  - Negative consequences in patients. According to some FPs, knowledge of the level and profile of risk can cause alarm or fear in some patients and even lead to the onset of depression and/or anxiety. *[FP64*: *Those who are very apprehensive*, *you tell them they are at moderate risk and they still spend the rest of their lives thinking*, *“Oh*, *I’m going to have depression"]*.
  - Negative attitude toward mental health. According to FPs there is a lack of motivation to address mental health problems in primary care professionals, especially in FPs *[FP5*: *FPs*, *although we are trained in mental health*, *we are generally reluctant to treat mental illness]*. Although they report that they receive training in mental health, this training may not be enough, which can cause discomfort and disinterest.
  - Changes in risk factors and lifestyle habits. Doctors noted the difficulty of changing patients’ habits and lifestyles. *[FP3*: *It is very difficult to change habits*, *lifestyles*, *it is easier to give a pill]*.
4. Facilitators perceived by the FPs.
  - The skills of the FP improved as he/she performed more visits-interventions with the patients, and also felt more comfortable if he/she knew the patient: *[FP34*: *The first ones (visits-interventions) may have cost me a little more*, *but since I knew these patients very well…]*.
  - Schedule management by the physician. *[FP22*: *…with proper organization*…*it was not an extra effort for me*…*as I can manage it*, *it was not a problem for me]*.
  - Physician training in communication skills. *[FP21*: *I have been working on communication for many years so in this regard it wasn’t difficult for me to inform them]*.
5. Positive consequences.
  - For physicians
    - The psychosocial approach was encouraged. This approach is used by FPs when they look at individuals in the context of the combined influence that psychological factors and the surrounding social environment have on their physical and mental wellness and their ability to function [15]. *[FP61*: *Giving them a space to address this issue allowed us to address the emotional side that we might not have addressed]*. Improves training in mental health and the management of people with psychosocial problems: *[FP32*: *Yes*, *the truth is that because of this I began to study depression a little more]*. *[FP62*: *Perhaps the project itself also gives you more resources for working with patients]*. The experience of FPs with the predictD intervention was perceived as an incentive to improve the psychosocial approach in primary care, increasing FPs’perception of self-efficacy when managing psychosocial problems of their patients.
    - Improves preventive care for depression. The physicians felt that the predictD intervention made them more alert to patients regarding primary prevention: *[FP24*: *For me it has been fundamentally about realizing that I have patients who are likely to have a depressive episode at a particular time and I hadn’t noticed them before]*. They even commented that it was useful to them for early diagnosis: *[FP10*: *The knowing the risk may be useful to you for an earlier diagnosis*…*]*.
    - It promoted a better understanding of the patients, it delved deeper into their lives and hidden problems could be discovered: *[FP30*: *You gave them a chance to tell you*.…*people open up more*…*you remove a little of the barrier of being on this side of the table]*.
    - Increases professional satisfaction and facilitates their work. *[FP59*: *It made things a little easier*, *so you were more aware*…*of doing a good job*. *The truth*…*it’s going to help me work better]*.
  - For patients
    - Improves mental health. From patients’ perspective, predictD intervention improved mental health. *[FP18*: *Patients appear more mentally healthy*, *those who have done the study*, *and more able to avoid falling into depression]*. These beliefs converged with the findings of our trial, which showed that the predictD intervention reduced the incidence of both depression and anxiety by 21% and 23% respectively with respect to the control group [7].
    - Promotes reflection, self-knowledge and awareness of the problem. *[FP32*: *I think that he was alerted to a series of things he was doing in his life that made it a little difficult for him to enjoy himself]*.
    - Activates resources in patients and empowers them. *[FP54*: *It makes him live life in a more positive way for himself*, *seeking out and recognizing the things he enjoys and enjoying them and removing a little bit of negativity that some people can experience]*.
    - Increases satisfaction. *[FP5*: *The patient is discussing a topic with you and at the same time he is also venting]*. *[FP16*: *People were excited*, *because they thought they saw that health professionals were showing more interest in their health]*.
  - For doctor-patient interaction
    Fosters a relationship of trust and understanding: *[FP54*: *…since then there has been a shift in the doctor-patient relationship in terms of more confidence and more empathy]*.
6. Future implementation of the predictD intervention. Some of the physicians have agreed to continue to use the predictD tool over time: *[FP61*: *I believe it is feasible because indirectly there are already many of us who do so]*. However, there were also conditions regarding its applicability: *[FP21*: *It is perfectly feasible*. *Everything will depend on the test (predictD risk algorithm)*, *if the test takes less than 5 minutes perfectly applicable*, *if the test takes 20 minutes it is not viable in Primary Care]*. Other physicians find it more difficult and suggest a number of modifications:
  - Intervene based on risk. *[FP60*: *What perhaps would not be useful is intervention for those at low risk*…*]*. Possibly having to carry out an intervention in all patients regardless of their level of risk (universal prevention) is perceived by FPs as an unrealistic workload. From this point of view, it would be more feasible to implement an intervention in a smaller number of patients, perhaps only with those at high risk (selective or indicated prevention). However, this assumption needs to be confirmed studying the effectiveness and cost-effectiveness in different profiles of patients.
  - Intervene in the most beneficial profiles. In this regard, no clear patient profile emerged in which the best results could be obtained. *[FP56*: *One of the patients who was always very negative*, *was always very depressed*, *perhaps it is not indicated for her]*. *[FP30*: *The patient who already had a predisposition (motivation)*, *he was much more receptive]*. *[FP62*: *In younger people perhaps it has been more useful]*.
  - Create a shorter, faster and simpler tool. *[FP60*: *It would have to be very short*, *very quick…to be done on a general level*. *Something very simple]*.
  - Incorporate the predictD tool into the clinical history. *[FP35*: *They should include it in the OMI (computerized health record) because if it is not in the OMI*, *it is not used]*.
  - Multidisciplinary intervention. Physicians believe that involving other primary care professionals may be helpful in implementing the intervention. *[FP5*: *We family physicians couldn’t manage that*, *it would have to be nursing*. *Nursing or other professionals]*.
  - Intervene in lengthier consultations. *[FP31*: *A little more time to give the patient the results*…*two or three more minutes]*.
  - Conduct workshops or support groups by risk levels and specific risks. *[FP64*: *In people at high risk and who would like to*, *hold psychological workshops]*. *[FP19*: *I would form groups of people with specific risks*…*that would allow you to change habits or introduce lifestyle changes]*.

**Fig 1:**
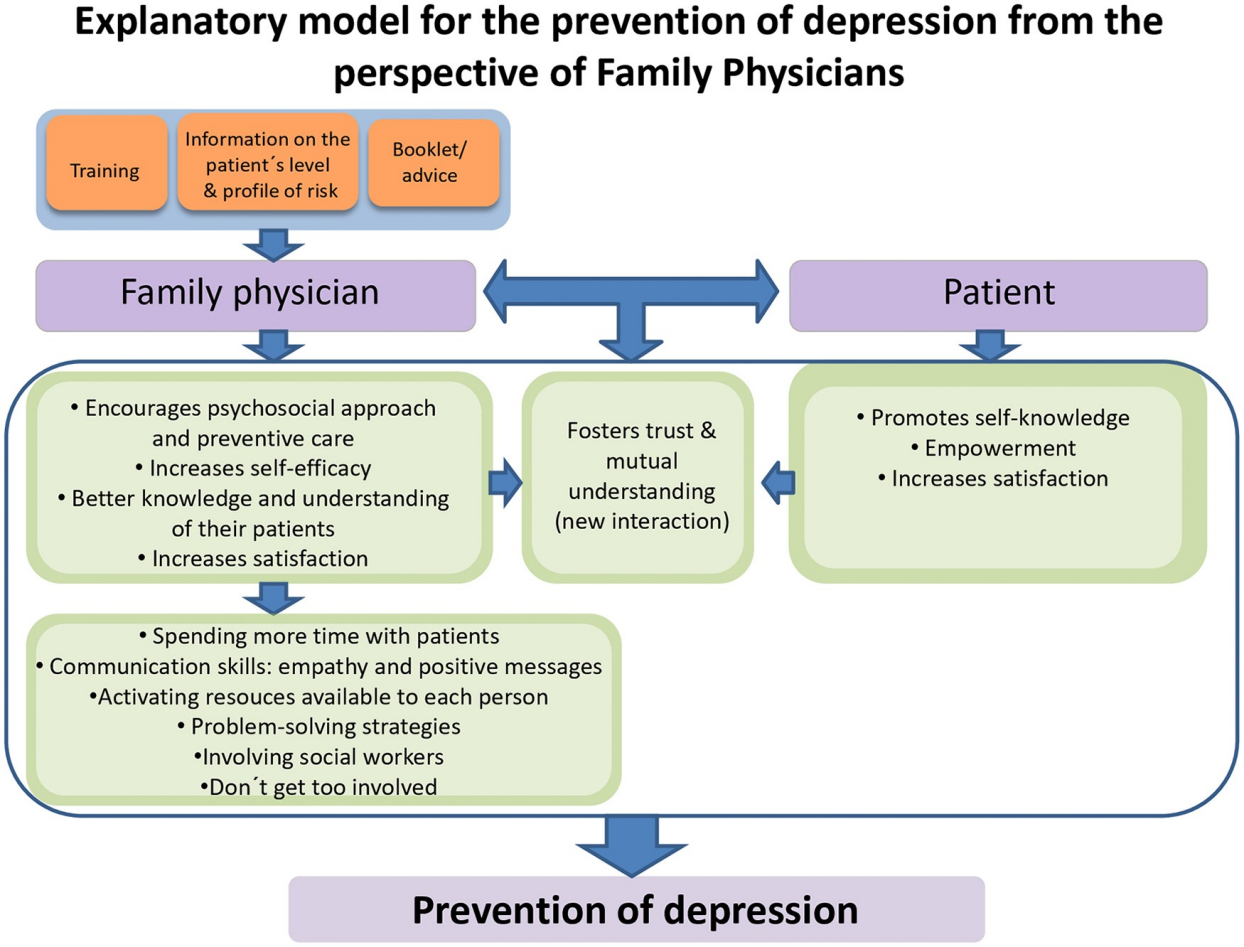
Explanatory model for the prevention of depression in primary care from family physicians´perspective.

The FPs perceived the information on the level and profile of risk of the patients, the brochure/advice and the training as mechanisms by which patients increased their self-knowledge, satisfaction and empowerment, therefore, improving their mental health. Additionally, these components fostered a relationship of trust and mutual understanding between the FPs and the patients and prompted the FPs to pay closer attention to the psychosocial aspects of their patients, favoring the knowledge of aspects of their patients, leading to increased satisfaction in the FPs. In consequence, the FPs spent more time with their patients, put into practice communications skills and problem-solving strategies, activated resources available to each person and involved social workers, while at the same time they tried not to get too involved themselves. All this, from the perspective of the FPs, resulted in the prevention of depression (Fig 1).

## Discussion

### Summary

This study reports on the opinions of the FPs concerning facilitators and barriers for the implementation of a personalized program, based on a risk algorithm, to prevent the onset of major depression in primary care. This information can be used to improve the predictD intervention, and to ease its adaptation and implementation to different settings. In general, the FPs had a positive experience with the predictD intervention, as it was easily embedded into their practice. It was perceived useful as a bio-psycho-social approach for improving the emotional health of their patients and their relationship with them, as well as their own satisfaction as a FP. However, they also detected some barriers such as lack of time, and the need for specific training to effectively communicate the risk of developing depression.

### Strengths and limitations

To our knowledge, this is the first study that explores the opinions and experiences of FPs who performed a personalized intervention to prevent major depression in primary care. The opinions of several of the FPs have been contrasted (see below) with the data available on the effectiveness and cost-effectiveness of the predictD intervention [7–8]. We emphasize that none of the FPs knew the results of the effectiveness or cost-effectiveness of the predictD intervention when they were interviewed for the predictD-EVAL qualitative study. The research team was multidisciplinary (family physicians, nurses, psychologists, psychiatrists and social workers), the approach was oriented, and the data were analyzed and interpreted from different professional perspectives thereby enriching the discussion and interpretation of the information. In addition, the data were triangulated for the elaboration of categories by three analysts. Respondent validation was conducted as part of a process of error reduction to establish the level of correspondence between researcher and research subjects.

There are also some limitations: First, some of the information derived from the FPs’ opinions would have been even more useful if it had been contrasted with the opinions of the patients. Even so, we know that most patients believed that the predictD intervention was acceptable (76%) and that patient adherence to the intervention was also good (90% of the patients attended at least two of the three planned intervention visits) [7]. Second, in some cases one year had elapsed from the time the FPs performed the interventions until they were interviewed. This time period may have been too long, affecting the memory of the FPs. Third, it is possible that the FPs gave more positive views about the intervention in order to satisfy the interviewers who were members of the research team for the study. Fourth, the FPs participating in this study possibly had a greater psychosocial and preventive orientation than the total of FPs because many FPs declined to participate in the trial [7]. Therefore the opinions of the FPs in this study would have limited its generalization. Most physicians tend to use a biomedical rather than a patient-centered communication style [16], and FPs with lower psychosocial orientation scores have poorer doctor-patient relationships [17].

### Comparison with existing literature

Previous acquaintance with patients and the doctor-patient relationship, the establishment of a basic psychotherapeutic relationship (not a psychiatric interview or formal psychotherapeutic interventions) [18], a family-oriented practice [19–20], social prescribing and community referral by FPs [21], and management of physical problems are all components of care or strategies commonly used by FPs in their clinical practice. The fact that FPs consider some aspects or components of the predictD intervention to be similar to what they already do in their routine practice favors their acceptance and implies a greater perception of self-efficacy in the role of the FP as facilitator in the predictD intervention and suggests a greater likelihood that they will be confident applying the predictD intervention. These are also strategies requested by primary care patients for prevention and the promotion of healthy habits [22].

In general, the FPs were satisfied with the training workshop for the predictD intervention; however, some pointed out the need for more extensive and continuous training. This might have been because a number of FPs had some difficulty in implementing the predictD intervention in the first consultations. The reduction in the onset of major depression attributable to the predictD intervention seemed to increase over time, which might be due to a dose–response effect of the intervention or simply a need for time and the accumulation of intervention visits to create the changes needed to prevent depression [7]. This would be in accordance with the opinion of the FPs regarding how their skills continued to improve as they had more visits-interventions with the patients [9]. Short-term training (less than 10 hours) is as successful as longer training Interventions for providers to promote a patient-centered approach in clinical consultations [23]. Perhaps an increase in training hours in the predictD intervention may not be necessary, but a review to improve the efficiency of training in those 10–15 hours is indicated or adding a booster after a given period.

FPs emphasized that the predictD intervention contributed to improving the doctor-patient relationship and patient confidence. Patients who gain a greater insight into their symptoms and feel understood by their physicians may be less anxious and depressive, have more confidence in their physician’s abilities, and be more trusting of their physician [24]. Furthermore, sharing of power and responsibility, the use of empathy, and treating the patient as a person all seem to be important FP communication strategies which help address barriers to completion of preventive services by patients [25].

Lack of time is a barrier widely reported by FPs, especially when undertaking preventive activities [17]. Some FPs considered that the predictD intervention generated a higher time and workload and others felt the opposite. In any case, objective data showed that the patients who received the predictD intervention, during the 18 months of follow-up, made fewer visits to the primary care centre than those in the control group, and this happened despite the patients in the intervention group having performed the three specific and mandatory visits (one every six months) of the predictD intervention [8]. In observational studies, the association between depressive and anxiety disorders and frequent primary care attendance has also been described [26–28].

Some FPs suggested that patients’ knowledge of their level and profile of risk of depression can cause alarm, fear and even the onset of depression or anxiety. However, the predictD intervention reduced the incidence of both depression and anxiety [7]. Moreover, in a previous study, primary care patients were asked if they would like to know their level and profile of risk of depression, and they generally displayed a positive attitude and were willing to be informed of their risk of depression [29]. In addition, FPs reported no adverse effects associated with the predictD intervention, and only three patients (0.0018%) in the intervention group contacted researchers, during the 18 months of follow-up, to complain about relationships with their FPs and to request a change of FP [7].

A number of FPs expressed some difficulty in getting their patients to understand their profile and level of risk for depression. That also happens, for example, in cardiovascular risk management [30]. How risk is communicated influences prevention [31], so improving clinicians’ skills to communicate it efficiently is a challenge that needs further evaluation.

The brochure delivered to patients was positively valued by FPs. The FPs may feel more secure in being able to provide information in a standardized way. However, it would have been useful to have collected the patients’ opinions in order to contrast this with the positive opinion of the FPs.

## Conclusion

The FPs suggested ways to adapt the predictD intervention to other settings and make its implementation more efficient. Some proposed prioritizing the ’constructing a tailored bio-psycho-family-social intervention by FPs to prevent depression’ component of the predictD intervention in patients at higher risk of depression. This is relevant to whether or the predictD intervention was more clinically and cost-effective in high or low-moderate risk patients. Other FPs suggested involving other primary care professionals (e.g. nurses) and minimizing the time to obtain information on the level and profile of the risk of depression. Our research team launched a free website (http://www.predictplusprevent.com/index.php?idioma=en) that anyone can access to calculate their risk of depression, anxiety or hazardous/harmful alcohol consumption. The website also provides information and resources on how to reduce risk. Some FPs, in addition to stepped care depending on the level of risk, also suggested specific interventions for patients with particular risk factors for depression (e.g. for sedentary or insomnia patients).

The predictD intervention requires little adaptation because it is based on usual components of primary care that could converge in the prevention of depression, and there now are validated risk algorithms in several European countries [32], the United States [33–35], Australia [36] and Latin America [37].

If depression prevention programs were massively scalable (e.g. through ICTs) and/or implemented in large populations (primary care, schools, and workplaces), they would have a considerable impact on patient health and quality of life and cost reduction, even were their effectiveness relatively low [1–3]. In this sense, a paradigm shift is needed [38]. This study provides key information on which to build an improved predictD intervention, enhancing factors considered useful by FPs and minimizing identified barriers. However, in order to achieve a more effective and acceptable intervention, the opinions of the primary care patients who received the predictD intervention are needed.

## Supporting information

S1 File Interview guide used in the study in Spanish. (PDF)
