# Supplementary material for: Family physicians’ views on participating in prevention of major depression. The predictD-EVAL qualitative study
Source: PLoS One. 2019 May 30;14(5):e0217621. doi: 10.1371/journal.pone.0217621 (PMC6542521; doi:10.1371/journal.pone.0217621)
Supplement: S1 File — (PDF) [file pone.0217621.s001.pdf]

CHECKLIST PREGUNTAS MÉDICOS

EXPERIENCIA CON LA PARTICIPACIÓN EL PROYECTO

Pregunta inicial general

- ☐ *¿Cómo ha vivido la experiencia de participar en este proyecto predictD?*

DAR INFORMACIÓN SOBRE LA PROBABILIDAD DE DEPRIMERSE EN EL FUTURO

Pregunta inicial general

- ☐ *A partir de su experiencia ¿Qué opinión tiene sobre dar a sus pacientes información sobre su probabilidad de deprimirse en el futuro?*

Dificultades para comunicar el nivel de riesgo:

- ☐ *¿Tuvo usted dificultades para informar a sus pacientes sobre su riesgo de deprimirse? ¿Piensa usted que los pacientes tenían dificultades para entender esta información?*

Comunicación de la información repetida en el tiempo:

- ☐ *¿Qué opinión tiene sobre la frecuencia con la que informaba a sus pacientes del riesgo de depresión (cada 6 meses)?*

Información sobre el perfil de riesgo:

- ☐ *Es posible que en algunos de sus pacientes usted intentara hacerles algún comentario sobre sus factores de riesgo para deprimirse. Si fue así, ¿Cómo fue su experiencia?*

Impacto emocional en los pacientes:

- ☐ *¿Cree usted que en algunos de sus pacientes el dar la información sobre su riesgo de deprimirse les pudo afectar emocionalmente? Si fue así, ¿Podría hablarme de ello?*

Impacto emocional en el profesional:

- ☐ *¿Cree usted que el dar la información a sus pacientes sobre su riesgo de deprimirse le afecto emocionalmente a usted miso? Si fue así, ¿Podría hablarme de ello?*

Mejoras:

- ☐ *¿Cambiaría usted algo sobre el contenido o la forma en la que usted informaba a sus pacientes sobre su riesgo de deprimirse?*

CONSEJOS Y FOLLETOS

Opinión sobre los consejos a los pacientes

- ☐ *Es posible que durante las entrevistas con sus pacientes de la intervención predictD usted tratara de aconsejarlos para prevenirles la depresión, ¿Qué opinión tiene de ello?*

Opinión sobre el folleto (enseñándole el folleto)

- ☐ *¿Qué opinión tiene sobre la utilidad del folleto para prevenir la depresión?, ¿Qué consejos del folleto le parecen más útiles?, ¿Cambiaría algo del folleto?*

UTILIDAD DE LA INTERVENCIÓN predictD

Opinión general sobre la intervención

- ☐ *¿Qué le parece la intervención predictD en cuanto a su utilidad prevenir la depresión en pacientes de atención primaria?*

Ingredientes activos

- ☐ *¿Qué ingredientes o partes de la intervención predictD cree que son más útiles para prevenir la depresión?*

Ingredientes prescindibles

- ☐ *¿Qué ingredientes o partes de la intervención predictD cree que son menos útiles para prevenir la depresión?*

Utilidad para el profesional

- ☐ *De la intervención predictD que usted ha implementado, ¿qué es lo que ha sido más útil para usted como profesional o para su actividad en su consulta?*

Utilidad para el paciente

- ☐ *De la intervención predictD que usted ha implementado, ¿qué es lo que ha sido más útil para sus pacientes?*

Utilidad en función de los pacientes

- ☐ *¿Cree usted que la intervención es más efectiva en un determinado perfil de paciente? ¿Cuál?*
- ☐ *¿Cree usted que la intervención predict no se debería implementar en un determinado perfil de paciente? ¿cuál?*

INTERACCIÓN

Cambio en las relaciones médico-paciente

- ☐ *¿Cambió en algo la relación con alguno de sus pacientes a partir de que le informara de su posibilidad de deprimirse en el futuro? Si fue así, ¿En qué aspecto o cómo cambió?*

PERCEPCIÓN DE EFICACIA

Percepción de eficacia de la intervención predictD

- ☐ *¿Cree usted que la intervención predictD que usted ha implementado a sus pacientes ha sido eficaz para prevenir la depresión? ¿En qué medida?*

DIFICULTADES

Percepción de barreras

- ☐ *¿Qué barreras o dificultades ha encontrado a la hora de desarrollar e implementar la intervención predictD?*

CAMBIOS Y MEJORAS

Sugerencia de cambios

- ☐ *Si usted volviera a realizar la intervención predictD con sus pacientes ¿Qué cambiaría?*

APLICABILIDAD FUTURA

Opinión general sobre la futura aplicación de la intervención predictD

- ☐ *En el caso en el que la intervención predictD fuese efectiva ¿qué piensa sobre su aplicabilidad futura de una forma generalizada como programa de prevención?*

Cambios en la práctica profesional a partir de la intervención predictD

- ☐ *¿Hay algo de la intervención predictD que esté empleando actualmente con sus pacientes? ¿Qué y por qué?*

Confianza en la aplicabilidad futura

- ☐ *Si la intervención predictD se aplicara de forma generalizada en el futuro. ¿recomendaría usted a sus familiares y conocidos que participaran en este programa de prevención?*

Sugerencias para la aplicabilidad futura

- ☐ *¿Se le ocurre alguna estrategia útil para la futura implementación de esta intervención como programa de prevención?*

ENTRENAMIENTO DE LA INTERVENCIÓN

Opinión general sobre el entrenamiento

- ☐ *Recordará qué antes de comenzar la intervención predictD usted participó en un taller de formación sobre la misma ¿Cuál es su opinión sobre el taller de formación para la intervención predictD?*

Contenidos

- ☐ *¿Qué opina de los contenidos del taller de formación para la intervención predictD?*

Método docente

- ☐ *¿Qué opina del método docente con el que se impartió el curso para la intervención predictD?*

Duración

- ☐ *¿Qué opina sobre la duración del taller de formación para la intervención predictD?*

Áreas de mejora

- ☐ *¿Qué cosas modificaría del taller de formación para la intervención predict?*

ENTRENAMIENTO DE LA INTERVENCIÓN

- ☐ *Ya estamos acabando la entrevista, ¿Desearía añadir algo más?*
